# Monthly Record of Deaths in the City of Buffalo

**Published:** 1859-01

**Authors:** H. D. Garvin

**Affiliations:** Health Physician


ART. VI.—*Report of Mortality in Buffalo for the Month of Nov.*, 1858
By H. D. Garvin, M. D., Health Physician.

| DISEASES. | No. | Males. | Females. | No Sex given. |
|---|---|---|---|---|
| Accident, | 2 | 2 | | |
| Aneurism, | 1 | 1 | | |
| Apoplexy, | 1 | 1 | | |
| Brain, Disease of, | 1 | 1 | | |
| Bronchitis, Chronic, | 1 | | 1 | |
| Cancer, | 1 | | 2 | |
| Cancer of Pancreas, | 1 | 1 | | |
| Cholera Infantum, | 1 | | 1 | |
| Convulsions, | 7 | 3 | 4 | |
| Croup, | 6 | 3 | 3 | |
| Cyanosis, | 1 | 1 | | |
| Delirium Tremens, | 1 | | 1 | |
| Dentition, | 2 | 1 | 1 | |
| Diarrhœa, Chronic, | 1 | | 1 | |
| Dropsy, | 2 | | 2 | |
| Dysentery, | 1 | 1 | | |
| Enteritis, | 2 | 1 | 1 | |
| Erysipelas, | 1 | 1 | | |
| Fever, Typhoid, | 3 | 3 | | |
| " Scarlet' | 2 | 1 | 1 | |
| " Gastric, | 1 | | 1 | |
| Hepatitis, | 2 | | 2 | |
| Hooping Cough, | 1 | 1 | | |
| Hydrocephalus, | 4 | 2 | 2 | |
| Heart, Disease of, | 4 | 1 | 3 | |
| Intemperance, | 1 | 1 | | |
| Lungs, Disease of, | 1 | 1 | | |
| " Congestion of, | 4 | 4 | | |
| Marasmus, | 4 | 2 | 2 | |
| Old Age, | 2 | 1 | 1 | |
| Peritonitis, | 2 | | 2 | |
| Phthisis, | 18 | 9 | 9 | |
| Pneumonia, | 6 | 5 | 1 | |
| Premature Birth, | 3 | 2 | 1 | |
| Pyemia, | 1 | | 1 | |
| Scrofula, | 1 | | 1 | |
| Ulceration of Mucous Membrane of Rectum, | 1 | | 1 | |
| Unknown, | 14 | 8 | 6 | |
| Total, | 115 | | | |

SEXES.

| | |
|---|---|
| Males, | 57 |
| Females, | 51 |
| Sex not given, | 7 |
| Total, | 115 |

AGES.

| | | | |
|---|---|---|---|
| Still-born, | 0 | Between 20 years and 30 years, | 6 |
| 1 day, | 4 | " 30 " " 40 " | 14 |
| 1 day and 30 days, | 4 | " 40 " " 50 " | 12 |
| Between 1 month and 6 months, | 7 | " 50 " " 60 " | 10 |
| " 6 months and 12 " | 10 | " 60 " " 70 " | 3 |
| " 1 year " 3 years, | 17 | " 70 " " 80 " | 2 |
| " 3 " " 5 " | 1 | " 80 " " 90 " | 1 |
| " 5 " " 10 " | 10 | " 90 " " 100 " | 0 |
| " 10 " " 20 " | 5 | " 100 " | 0 |
| | 58 | | 48 |
| Ages not given, | 9 | | 58 |
| Total, | 115 | | |

NATIVITIES.

| | | | |
|---|---|---|---|
| American, | 66 | French, | 1 |
| German, | 18 | Holland, | 1 |
| Irish, | 19 | Wales, | 0 |
| Canadian, | 1 | Prussian, | 0 |
| English, | 2 | Italian, | 0 |
| Scotch, | 1 | Country not given, | 6 |
| Total, | | | 115 |

---

*Anœphrodisiac Properties attributed to Belladonna.*—Dr. J. F. Huestis of Mobile, Ala., in a letter to Dr. B. Dowler, of the *New Orleans Medical and Surgical Journal*, states that while giving belladonna to a gentleman afflicted with whooping cough, the patient noticed that, during the whole time he was taking it, " he was unable to accomplish even an erection." He suggests the applicability of this article in case of chordee. He had himself used it with perfect success in a case of distressing nocturnal emissions.

